# Machine Learning Models and Statistical Complexity to Analyze the Effects of Posture on Cerebral Hemodynamics

**DOI:** 10.3390/e24030428

**Published:** 2022-03-19

**Authors:** Max Chacón, Hector Rojas-Pescio, Sergio Peñaloza, Jean Landerretche

**Affiliations:** 1 Departamento de Ingeniería Informática, Universidad de Santiago de Chile, Av. Víctor Jara N° 2659, Estación Central, Santiago 9190864, Chile; hector.rojas.p@usach.cl (H.R.-P.); sergio.penaloza@usach.cl (S.P.); 2 Unidad de Neurología, Escuela de Medicina, Universidad de Santiago de Chile, Av. Alameda N° 3336, Estación Central, Santiago 9170022, Chile; jean.landerretche@usach.cl

**Keywords:** cerebral hemodynamics, statistic complexity, machine learning models, postural effects

## Abstract

The mechanism of cerebral blood flow autoregulation can be of great importance in diagnosing and controlling a diversity of cerebrovascular pathologies such as vascular dementia, brain injury, and neurodegenerative diseases. To assess it, there are several methods that use changing postures, such as sit-stand or squat-stand maneuvers. However, the evaluation of the dynamic cerebral blood flow autoregulation (dCA) in these postures has not been adequately studied using more complex models, such as non-linear ones. Moreover, dCA can be considered part of a more complex mechanism called cerebral hemodynamics, where others (CO_2_ reactivity and neurovascular-coupling) that affect cerebral blood flow (BF) are included. In this work, we analyzed postural influences using non-linear machine learning models of dCA and studied characteristics of cerebral hemodynamics under statistical complexity using eighteen young adult subjects, aged 27 ± 6.29 years, who took the systemic or arterial blood pressure (BP) and cerebral blood flow velocity (BFV) for five minutes in three different postures: stand, sit, and lay. With models of a Support Vector Machine (SVM) through time, we used an AutoRegulatory Index (ARI) to compare the dCA in different postures. Using wavelet entropy, we estimated the statistical complexity of BFV for three postures. Repeated measures ANOVA showed that only the complexity of lay-sit had significant differences.

## 1. Introduction

Currently, three mechanisms are recognized in cerebral hemodynamics, namely: dynamic Cerebral Autoregulation of blood flow (dCA), the cerebral blood flow reactivity to CO_2_ and cerebral neurovascular-coupling (NVC).

dCA is a cerebral homeostatic mechanism that maintains relatively constant cerebral blood flow (BF) despite the changes that may be produced by arterial blood pressure (BP). One way to measure dAC is to determine the transcranial flow output (capturing with Transcranial Doppler, TCD) in response to a negative BP step [1,2].

The classic method for producing a BP drop was proposed by Tiecks et al. [3], induced by the sudden release of compressed bilateral thigh cuffs. A number of other maneuvers have been proposed to induce changes in BP, to provoke a corresponding response in BFV [4,5,6]. However, the most interesting are those that imitate natural body movements, such as posture changes. For these, there are a considerable number of studies that use them [7,8,9,10,11,12,13,14,15,16]. One significant advantage is that they are simple to use in the elderly and pregnant women. Through their use, some studies prove that changes in posture do not affect the calculation of the dCA [17,18,19,20,21], but they all use indices and linear models. Of note is the work of Mahdi et al. [21] where they test four different indices to obtain dCA and two different linear models to calculate it.

However, there is a clear question regarding the linearity of dCA [22,23,24], which has led to the emergence of non-linear methods to assess dCA [25,26,27,28,29,30,31,32]. From the point of view of hemodynamics, dCA is not an isolated mechanism; on the contrary, there are complex interactions that include mechanisms of reactivity and neurovascular coupling. The experiments [26,27,32] proved the non-linear interaction between reactivity and dCA. This generates the necessity to evaluate the behavior of this system, in terms of the different postures, in a more comprehensive way, considering its complexity and interaction.

Through nonlinear modeling of dCA using SVM and the study of complexity based on wavelet entropy, applied to the same set of experimental data, we believe that it is possible to describe the multiple interactions and differences produced in cerebral hemodynamics when making usual body posture changes in healthy humans.

## 2. Materials and Methods

### 2.1. Subjects and Measurement

Eighteen young adult volunteers were recruited at Universidad de Santiago de Chile, without a history of diseases or cardiovascular problems, hypertension or diseases of the nervous system, with average age of 27.00 ± 6.29 years (range 22 to 44, approved by the ethics committee of the University, 22 January 2016). The BFV was monitored in the middle cerebral artery using a transcranial Doppler-Box brand DWL, with two ultrasonic transducers of 2 MHz, to obtain the arterial BFV in both cerebral hemispheres. The arterial blood pressure was monitored with a non-invasive continuous pressure monitor, Finapres finometer MIDI, in the middle finger of the non-skillful hand. Each signal was sampled at 100 Hz and stored directly on a computer using the Doppler-Box system’s management software. 

After setting the ultrasonic transducer and the Finapres, the subject was maintained at rest for five minutes. Then, the order of the postures to be evaluated was chosen randomly (stand, sit, lay). Each posture was held for five minutes with an interval of three minutes to change posture and stabilize.

The manual repair of significant pieces of signal (greater than 4 samples) using cubic spline to interpolate these values was required. Then, automatic preprocessing software removed noise with two different filters. The Hampel filter was used to eliminate outliers (this filter works over median values). The Butterwort low-pass filters are of the eighth order and have a cutoff frequency of 20 Hz. This filter was applied using two filters of fourth order in push–pull to achieve phase zero. Then, heartbeat detection was performed (locating the upstroke, max, and min points in BP). With these values on each beat, the average values of the BP and BFV signals were calculated. These were interpolated and samples were taken at a constant rate of 0.4 samples per second.

Finally, the normalization of the signal was carried out using the min–max feature scaling method (with a range of [−1, 1]) shown in Equation (1), to be used in the calculation of the ARIs indices and the non-linear models with the SVM.

(1) $$x_{norm}\left(n\right)=2\frac{x\left(n\right)-x_{min}}{x_{max}-x_{min}}-1$$

where, $x_{norm}$ denotes normalized value of *x*, $x_{min}$ and $x_{max}$ are, respectively, the minimum and maximum value of the signal.

### 2.2. Methods

#### 2.2.1. Machine Learning Models of dCA

Support Vector Machines have been used to obtain non-linear models of the BP–BFV dynamic relationship and the foundations of SVM are presented in extended form in our work [32]. Once these models are trained, it is possible to use a step-change in BP as an input and then obtain the BFV response as an output, from which estimates of ARI can be derived, following the procedure described in Appendix A.

SVMs are static models that relate an input vector to an output real value. To capture the temporal relationship of two or more signals, it is necessary to add external delays or recurrences. Specifically, we used ν-SVR [33] to develop dynamic univariate models with BP as input and BFV as an output of two types: non-linear Finite Impulse Response (NFIR) models and non-linear Autoregressive with eXogenous input (NARX) models. To obtain linear models, it is enough to transform the nonlinear function f into a linear combination of its arguments to obtain models FIR and ARX. The static SVM solves an optimization problem that is presented in [33], and its objective function is included in Equation (2).

(2) $$minimize\;\theta \left(\vec{w},\;\xi \right)=\frac{1}{2}{\left\|\vec{w}\right\|}^{2}+C\left(l\nu \varepsilon +\overset{l}{\underset{i=1}{\sum }}\xi_{i}\right)$$

where $\left\|\vec{w}\right\|$ stands for the Gaussian kernel function shown in Equation (3).

(3) $$\left\|\vec{w}\right\|=K\left({\vec{x}}_{i},\;{\vec{x}}_{j}\right)=\exp\left(\frac{-\left\|{\vec{x}}_{i}-{\vec{x}}_{j}\right\|{}^{2}}{2\sigma^{2}}\right)$$

where C, ν and ε correspond to SVM hyper-parameters.

To implement the dynamic model of the SVM on the BP signals (*p*(*n*)) and predicted BFV (*v^^^*(*n*)) Equations (4) and (5) are used, where *n*, *n_p_* is the delay in the BP signal, *n_v_* corresponds to BFV recurrences, and *f* represents a non-linear function. Estimates for kernel radial basis function of SVM shown in Equation (3).

*v*^^^(*n*) = *f*(*p*(*n*), *p*(*n* − 1), …, *p*(*n* − *n*_*p*_)) (4)

*v*^^^(*n*) = *f*(*v*^^^(*n* − 1), *v*^^^(*n* − 2), …*v*^^^(*n* − *n_v_*), *p*(*n*), *p*(*n* − 1), …, *p*(*n* − *n_p_*)) (5)

Each pair of signals, i.e., simultaneous recordings of spontaneous fluctuations of BP and BFV, were separated into two sections: the training segment, consisting of the first two and a half minutes of the signals, and the validation segment, with the last two and a half minutes. All models were trained with the former section using the one-step-ahead prediction strategy, and validated with the latter fragment following the model predictive output strategy, in which the model predicts the complete BFV validation segment for the unseen validation BP segment, resulting in balanced cross-validation [34]. The search for delays in both the BP signal (*n_p_*) and the BFV recurrences (*n_v_*) was conducted empirically. The hyper-parameters of non-linear ν-SVR (i.e., *C*, *v*, ε and *σ*) were bounded by grid search. 

The efficiency of a model was determined by Pearson’s correlation coefficient (CC) between the real BFV (*v*) signal and the BFV (*v^^^*) estimated by the model. The best model for each subject is the one with the highest CC in the validation segment. However, high correlation is not enough to guarantee that a model’s response has physiological plausibility. To solve this problem, we implemented a computational routine based on the indications suggested by Ramos et al. [35], where the physiological quality is evaluated according to the response of the BFV to negative BP step, using three simple criteria: (i) the response should be reduced to at least 40% of the original level from the BFV signal’s mean level; (ii) the response return must be between the minimum and average value plus 10%; and (iii) the BFV return must be between 3 and 6 s after the pressure drop. That automatically discards models that generate non-physiological responses. Training and validating subroutines were implemented using the R environment [36] and libsvm [37] in package e1071 [38].

#### 2.2.2. Statistic Complexity of Hemodynamics

The Wavelet transform, like the Fourier transform, uses an orthogonal basis to decompose the signal in a unique and invertible way. Bases such as Biorthogonal, Daubechies, Haar, and Symlets, among others [39], correspond to the oscillating basis of fast and smooth extinction.

For the calculation of the wavelet entropy, we used the implementation presented by Rosso [40]. Using discrete time functions $\psi_{j,k}\left(n\right)$, which decompose the signal into different dilation (*j*) and translation (*k*) scales representing different frequency levels, both expressed in powers of two. As shown in Equation (6), they allow decomposing of the BFV signal (*v*(*n*)) into a linear combination of these functions and coefficients $C_{j}\left(k\right)$, similar to the Fourier series.

(6) $$v\left(n\right)=\overset{-1}{\underset{j=-N}{\sum }}\underset{k}{\sum }C_{j}\left(k\right)\psi_{j,k}\left(n\right)\;$$

**Wavelet distribution.** This discrete transform represents the signal *v*(*n*) in its coefficients $C_{j}\left(k\right)$. Since the ψ functions turn out to be an orthogonal basis, the energy of each scale *j*, the sum of the squared coefficients, at the different frequency levels *j* = *1*…*N* results in the energy for each resolution level *j*, Equation (7).

(7) $$E_{j}=\underset{k}{\sum }{\left|C_{J}\left(k\right)\right|}^{2}$$

The total energy is obtained by summing over all scales. An adequate approximation of the probability distribution of the signal can be obtained by calculating the ratio between the energy of each scale, over the total energy, obtaining $\left\{p_{j}\right\}$.

In this way, entropy characterizes a phenomenon that represents the signal in time and frequency (or scale).

**Entropy and complexity.** The total entropy of the signal will correspond to the classical definition given by Shannon et al. [41], which is obtained in this case from the relative wavelet energy. A measure of the information contained in the signal, Rosso et al. [40] Equation (8):

(8) $$H=-\overset{m}{\underset{j=1}{\sum }}p_{j}ln\left(p_{j}\right)$$

For statistical complexity, we used Lopez-Ruiz’s definition, based on the disequilibrium (*Q*) of the system [42,43,44,45]. This is obtained as the distance between the uniform distribution $P_{e}$ (maximum entropy) and the distribution of the signal at issue $P_{s}$, Equation (9).

(9) $$Q=Q_{0}D\left(P_{e},P_{s}\right)$$

where *D* corresponds to Euclidean distance or Wooters’ distance defined as:

(10) $$Q=Q_{0}cos^{-1}\left\{\overset{m}{\underset{j=1}{\sum }}{\left[p_{j}\right]}^{1/2}{\left[\frac{1}{m}\right]}^{1/2}\right\}$$

with $Q_{0}=m/\left(m-1\right)$ for Euclidean distance and $Q_{0}=1/cos^{-1}\left\{{\left[1/m\right]}^{1/2}\right\}$ to Wooters’ distance [46].

In this way, statistical complexity is defined as the product of entropy and disequilibrium.

(11) C=HxQ

**Blood flow velocity analysis.** Many times, the value of the complexity represented by the different states is enough to apply the statistical tests directly to the complexities of the analysis. However, Equation (11) shows a compound characteristic that considers entropy, but its dependence is not direct since the imbalance is added as a product.

We will use complexity to perform the statistics, but we will rely on a graphical representation called the entropy complexity plane [47], where the normalized entropy (in the interval [0–1]) is shown on the abscissa axis in an increasing way and the complexity on the ordinate axis.

This plane is of great interest not only because it contributes an additional dimension, but also because in the case of biological systems. Substantial evidence shows there is a decrease in complexity [48] in the presence of disease or aging, which can be better appreciated on this representation.

### 2.3. Statistical Analysis

Data normality was tested using Shapiro and Wilk’s statistics. Paired comparisons for cerebral hemispheres were made with the Student’s t-test in normality and with the Wilcoxon test when normality was rejected. When differences in hemispheres didn’t exist, indices or complexities were averaged.

We use repeated measures ANOVA analysis for comparison indices and complexities [49]. The discriminatory ability of the different approaches was compared in terms of the area under the ROC curve (AUC). ROC curves were obtained from autoregulation index values by using numbers in [0–9] for the SVM models and complexity analysis. AUC values are achieved by each method [50]. In all cases, *p* < 0.05 was considered statistically significant.

## 3. Results

One subject was discarded since the BFV of one position was unrecoverable. Four of the remaining BFVs required manual spline repair. The rest of the BFV and BP signals were easily fixed by using Hampel and Butterwort filters.

After applying the normality tests, there were no significant differences between both cerebral hemispheres for any subject. The results of the separate processes of each hemisphere also occurred without significant differences. Therefore, complexity and ARIs’ values were always averaged as a single value for each subject.

The average values for the eighteen subjects are shown in Table 1.

**Figure 1:**
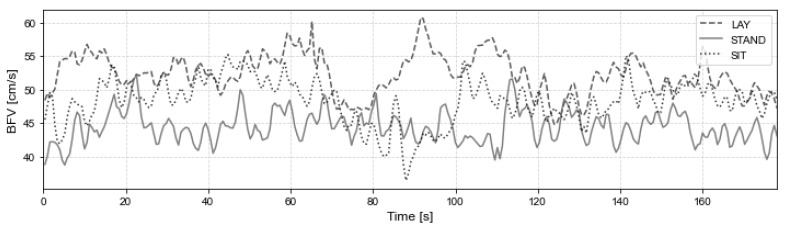
BFV signal for subject #12, in three postures, lay (dashed line), stand (solid line) and sit (point line).

Of the signals in all three postures, only the signal in the lying position had a slightly higher amplitude, while the others had similar characteristics in amplitude and frequency. The three BFV signals for a typical subject are presented in Figure 1.

### 3.1. SVM Models

The parameters and results for the four models (FIR, NFIR, ARX and NARX) are shown in Table 2. The ranges for the input and output signal delays and hyperparameters of the SVM are shown. Correlation values are also displayed in the evaluation segment.

With the BFV step response, it is possible to apply the method of Appendix A (classic ARI index) to obtain a dCA index for each subject. The average values for each model and posture are presented in Table 3. Non-significant differences exist in three positions in any model.

### 3.2. Statistic Entropy and Complexity

The Shapiro and Wilk’s test of the complexities turned out to be normal, and the paired tests of the cerebral hemispheres did not present significant differences.

The ANOVA test is *p* = 0.0059 (F statistic = 5.9807, DF = 2) and Tukey’s post hoc test shows that the differences between Sit and Lay are 0.0023, this is 22 times less, than the limit (*p* < 0.05) set as statistically significant for biological studies. The obtained *p*-values for the differences between Lay and Stand and Sit and Stand were 0.1418 and 0.2406, respectively.

**Figure 2:**
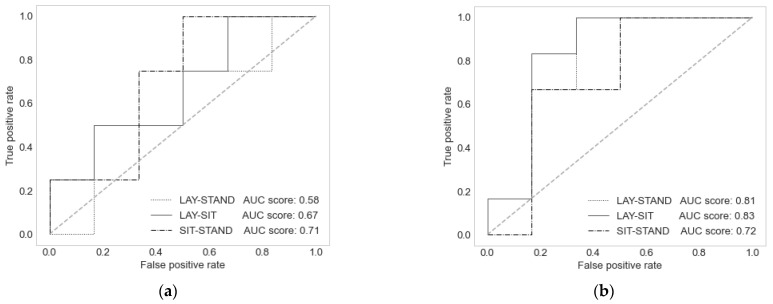
ROC curves to classify paired comparisons of the three postures. (**a**) Among the SVM-NARX model postures’ dCA values. (**b**) Among the BFV postures’ complexities.

As a way to quantify the classifying power of the methods, we calculated the ROC curves and their area under the curve (AUC). Figure 2a shows the ROC curves for the best model in terms of fit (NARX, correlation) for the comparison between the three postures: sit–stand, lay–sit, and lay–stand. For its part, Figure 2b shows the ROCs for the same comparisons among complexities. The largest AUC in the area corresponds to the differences between the lying and sitting postures in Figure 2b.

**Figure 3:**
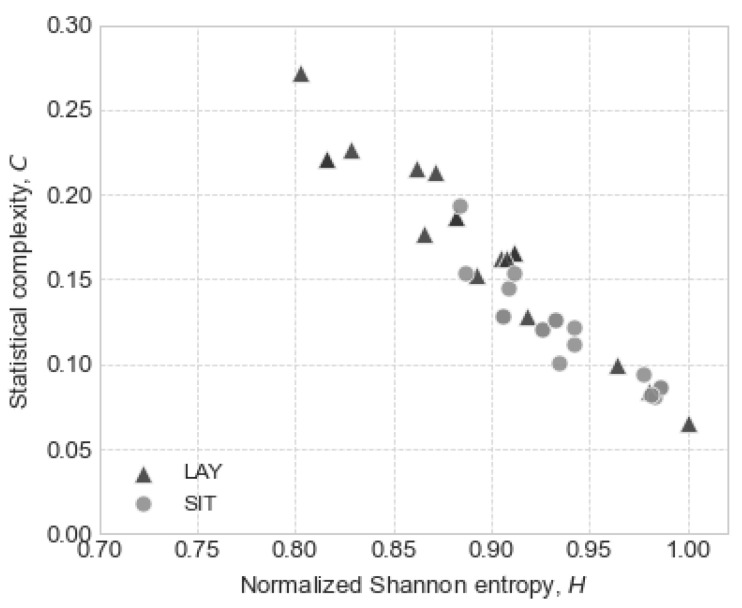
Complexity–entropy plane for lay–sit comparison. Subjects represented by circles correspond to the sitting posture, and subjects represented by triangles represent the lying posture.

Finally, as an aid for the results analysis and to better quantify both the complexity and the entropy of the postures, we show the distribution of the subjects in the complexity–entropy plane (Figure 3), the only comparison that turned out to be significantly different.

## 4. Discussion

We chose to use SVM methods over time, due to their determinism to train them, versatility, and power to analyze multiple variables [32], which allows explaining the interaction with reactivity in great detail. It also allowed us to easily turn it into a linear model.

**Figure 4:**
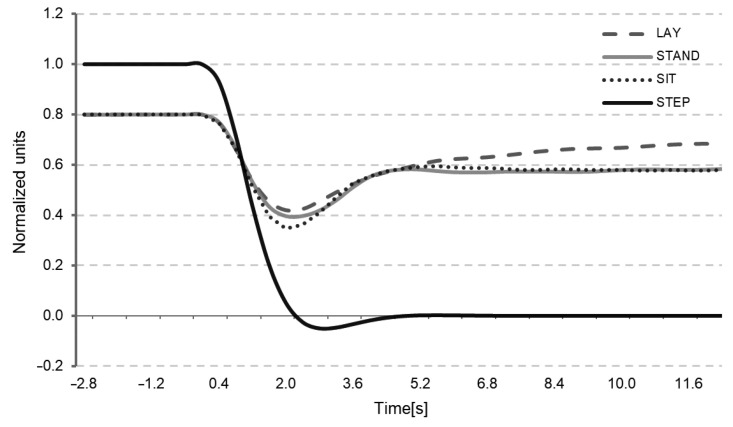
Average NARX estimated BFV response to negative BP step (solid black line), lay (dashed line), stand (solid grey line), and sit (pointed line).

These models allowed for the estimation of autoregulation via the BFV’s responses to changes in BP (Figure 4). By performing an ANOVA analysis for both linear and non-linear models, the values of the autoregulation response were not significantly different from the responses of the BFV for posture.

Current evidence from studies that attempted to link postures to dCA [17,18,19,21] indicates that there are no differences in autoregulation based on posture. It should also be noted that these works were implemented using a linear perspective.

In the literature, the work that presents a reasonable doubt is [20] where the squat-to-stand maneuver is studied. The results show that, when considering the direction of the maneuvers (that is, squat-to-stand and stand-to-squat), the results are not the same, and these effects are explained by concomitant changes in the BFV and pulse pressure. However, it is not possible to establish a significant difference in autoregulation when the maneuvers are performed repeatedly. This also proves the relationship of non-linearity, since the asymmetry in directionality is proof of it.

Like this, this work coincides with our findings. Although the phenomenon has non-linear characteristics, the postures do not affect the calculation of the dCA, whether it is carried out linearly or not. Despite the previous coincidence, suspicions rose in [20] regarding the incidence of other physiological conditions or effects of age, sex, or pathologies, which remain completely valid. 

To obtain an answer, an experimental design that includes these variables or effects and uses a non-linear technique such as multivariate SVMs could be proposed.

Our solution was to use a tool that not only considers the nonlinearity of the phenomenon, but also its complexity with the same data from this experiment, and that can detect other characteristics in the BFV, such as variance over time or discover more sensitive aspects such as co-variables, which do not necessarily have to be explicitly identified.

**Figure 5:**
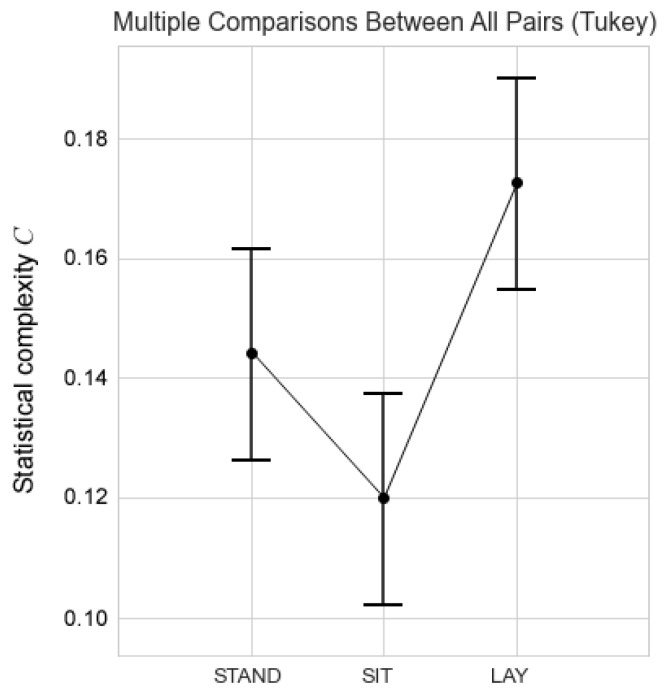
The graph shows the universal confidence interval of the means for each posture.

The verification of these differences is shown through the analysis of repeated measures ANOVA, for the complexities of the 3 postures, which presents a *p*-value of 0.0059, and their differences are observed in Figure 5. However, the post-hoc analysis of Tukey only shows significant differences between complexities for lying and sitting, with a *p*-value of 0.0023. These findings are significant in comparison to the current literature [18,19,21] because none of these studies show significant differences in cerebral autoregulation in changes of posture.

An interesting graphic representation that can shed light on our findings is the so-called complexity–entropy plane [51]. Although the cases of the lying posture seem to cross the entire section of the plane where the two maneuvers are deployed, starting from high complexity and low entropy, to low complexity and higher entropy, in fact, a majority of these cases have a higher complexity. In contrast, sitting cases can be more clearly identified with the lower right quadrant, which represents a lower complexity and greater disorder.

When analyzing BFV, a hemodynamic phenomenon should be considered rather than just dCA, although the dCA component should be the majority since the factors are relatively constant, such as neurovascular coupling or CO_2_ variation.

We think that the significant differences in complexity are due to a more intrinsic cause of the system. The results show that the state of rest leads to an increase in complexity. From a system utilization point of view, this state could represent a lower metabolic demand, not having to control posture as it would be in the other two cases.

The question to be elucidated here is whether a higher metabolic demand decreases the complexity. The work of Zarjam et al. [52] shows (with the same wavelet entropy calculated in this work) that it is possible to associate low entropy states as the mathematical tasks become more complicated. Furthermore, in our work [53], we calculated the complexity (using multi-scale entropy) in the case of hearing sounds with emotional content, such that the state of lower complexity is the state when listening to music with strong positive emotional content for the subject. We associated this with a greater demand on the cerebrovascular system.

From a more general perspective, complexity measurement is certainly a much more general mechanism for assessing brain function than the non-linear modeling of dCA.

Clearly, complexity is constituted as a powerful biomarker for differences, not only between pathologies but also between mental states or different conditions, whether these are pathological or different states of health, such as ageing [48].

These findings can also be seen in a recently published work [54], which studies a large number of subjects with epilepsy (100) and their control cases, but manages to classify the subjects according to different health conditions, such as metabolic disorders and others, which were not considered as the initial underlying pathology.

The limitations of this study are the same as any other using a sensitive diagnostic tool for a wide spectrum of alterations, its lack of specificity. This is despite the fact that we have a clear hypothesis to explain the increase in complexity found in the lay posture. To appropriately explain the differences found in complexity, using a non-linear model where the neurological control of posture could be measured would be a definitive test.

## 5. Conclusions

We have shown that the different postures of the human body do not affect the calculations of brain blood flow autoregulation, even when this system has non-linear behavior. However, we have also been able to observe that, when considering the cerebral hemodynamic system from a more comprehensive perspective (beyond the BP–BFV relationship), it is possible to see that the postures do affect this system. Differences were found thanks to the application of statistical complexity, using a highly sensitive dynamic systems analysis tool. However, the specificity of this technique still requires further study.

## Figures and Tables

**Table 1:** Mean ± SD of BP in mm [Hg] and BFV in [cm/s] for hemisphere and average in three postures.

Posture	Lay				Stand				Sit			
Signal	BFV			BP	BFV			BP	BFV			BP
Side	Right	Left	Mean	-	Right	Left	Mean	-	Right	Left	Mean	-
Mean	58.52	60.02	59.27	74.96	54.36	52.65	53.50	84.81	53.69	52.07	52.88	81.45
Std	13.48	14.23	13.68	9.03	11.47	10.31	10.79	10.93	13.50	11.22	12.26	11.87
CoV	0.23	0.24	0.23	0.12	0.21	0.20	0.20	0.13	0.25	0.22	0.23	0.15

**Table 2:** Hyperparameters and fit indices for SVM models.

Model	n_p_	n_v_	C	v	σ	CC Lay	CC Stand	CC Sit
FIR linear	[1–10]	-	[−2, 14 e^inf^]	[0, 1–0, 9]	[−1, 5]	0.611	0.721	0.626
FIR non-linear	[1–10]	-	[−2, 14 e^inf^]	[0, 1–0, 9]	[−1, 5]	0.655	0.742	0.682
AR linear	[1–8]	[1–6]	[−2, 14 e^inf^]	[0, 1–0, 9]	[−1, 5]	0.553 *	0.706	0.599
AR non-linear	[1–8]	[1–6]	[−2, 14 e^inf^]	[0, 1–0, 9]	[−1, 5]	0.749	0.809 *	0.761

* Most statistically significant differences *p*-value = 0.005.

**Table 3:** The results of the application of the repeated measures ANOVA methods on the ARIs’ values, for the four models.

Model	Lay	Sit	Stand	*p*-Values ANOVA
FIR ARI	4.69 ± 2.51	3.6 ± 2.15	4.76 ± 2.23	0.2522
NFIR ARI	4.41 ± 1.94	4.12 ± 2.58	4.97 ± 2.03	0.3201
ARX ARI	4.41 ± 2.57	4.25 ± 1.72	4.42 ± 1.92	0.9683
NARX ARI	4.92 ± 2.31	3.84 ± 2.28	4.51 ± 2.77	0.6991

## Appendix A. Classic Dynamic Autoregulation Index

The Aaslid and Tiecks’ ARI index [1, 3] is the most widespread linear method in the autoregulation literature, which evaluates the changes in the BFV in response to the changes introduced in the BP by the sudden release of the cuffs of the inflated bilateral thighs.

The ARI index classifies subjects on a scale from 0 to 9, corresponding to 10 different ARI values (quality of autoregulation), where 0 represents the complete absence of autoregulation and 9 is considered good autoregulation. This method estimates the BFV using a second-order linear differential equation that uses two state variables, *x*_1_ and *x*_2_, as shown in the equations in Equation (A1).

(A1) $$\begin{array}{c} NP(t)=\frac{p(t)}{1-CCP};\\ x_{1}(t)=x_{1}(t-1)+\frac{NP(t-1)-x_{2}(t-1)}{f\times T}\\ x_{2}(t)=x_{2}(t-1)+\frac{x_{1}(t-1)-2\times D\times x_{2}(t-1)}{f\times T};\\ \hat{v}(t)=1+dP(t)-K\times x_{2}(t) \end{array}$$

where *f* is the sampling frequency and *CrCp* refers to the Critical Closing Pressure that can be calculated from the initial BP and BFV. *NP(t)* corresponds to the change of the normalized BP from a control value. The model parameters are given by *K, D* and *T*, where *K* is a parameter that represents the autoregulating gain of the system, *D* is a damping factor, and *T* is a time constant. The relationship established between parameters and ARI values is shown in Table A1.

**Table A1:** Relationship between Equation (6) parameters and the ARI index.

T	D	K	ARI
2.00	1.70	0.00	0
2.00	1.60	0.20	1
2.00	1.50	0.40	2
2.00	1.15	0.60	3
2.00	0.90	0.80	4
1.90	0.75	0.90	5
1.60	0.65	0.94	6
1.20	0.55	0.96	7
0.87	0.52	0.97	8
0.65	0.50	0.98	9

For each produced maneuver at *p*(*t*), a corresponding velocity *v*^^^(*t*) is predicted from the model, which can be compared with the real speed *v*(*t*), and whose highest correlation represents the model’s quality (lowest error).

The model predicts a corresponding velocity *v*^^^(*t*) for each produced maneuver at *p*(*t*), which can be compared to the real speed *v*(*t*), and whose highest correlation represents the model’s quality (lowest error).

To implement this method in the SVM model, we replace and maneuver the changes introduced in the BP by the sudden release of the cuffs of the inflated bilateral thighs, for a negative step response to the BP, as shown in Figure 2, which are adjusted by Equation (A1) and the ARI value obtained by interpolation of the values (*K*, *D* and *T*) of the fitted equation on Table A1.
